# Targeted Amino-Terminal Acetylation of Recombinant Proteins in *E. coli*

**DOI:** 10.1371/journal.pone.0015801

**Published:** 2010-12-23

**Authors:** Matthew Johnson, Arthur T. Coulton, Michael A. Geeves, Daniel P. Mulvihill

**Affiliations:** School of Biosciences, University of Kent, Canterbury, United Kingdom; Baylor College of Medicine, United States of America

## Abstract

One major limitation in the expression of eukaryotic proteins in bacteria is an inability to post-translationally modify the expressed protein. Amino-terminal acetylation is one such modification that can be essential for protein function. By co-expressing the fission yeast NatB complex with the target protein in *E.coli*, we report a simple and widely applicable method for the expression and purification of functional N-terminally acetylated eukaryotic proteins.

## Introduction

In eukaryotic cells up to 98% proteins are N-terminally acetylated [1]. In many of these cases the acetylation is required for normal function e.g. α-crystallin, S-adenosylmethionine decarboxylase, thymosin and components of the 26S proteasome regulatory particle [2], [3], [4], [5]. In others N-terminal acetylation is a regulatory event e.g. fission yeast tropomyosin (Tm), Cdc8 [6]. In all cases the acetylation stabilises the protein by protecting it from degradation via N-terminal proteases.

How acetylation is brought about and how it changes protein function is poorly understood. Amino terminal acetylation within eukaryotes is carried out by N-α-acetyltransferase (Nat) complexes and is thought to take place co-translationally at the ribosome [7]. Currently 3 distinct classes of Nat complexes have been identified (NatA, B & C) [8], each composed of distinct catalytic and regulatory subunits. These complexes associate with specific and distinct target sequences at the amino terminus of elongating polypeptides.

The lack of Nat complexes or their equivalents within prokaryotes has prevented the use of *E. coli* expression systems for producing N-terminally acetylated proteins. Although gram-negative bacteria, such as *E. coli*, are capable of acetylating components of their own proteome, it occurs infrequently and is undertaken by a discrete molecular pathway. The inability to produce N-terminally acetylated eukaryotic proteins in *E. coli* limits the ability to generate low cost proteins & peptides. Researchers are currently dependent upon the use of comparatively expensive and time-consuming chemical acetylation or eukaryotic expression systems to complete functional studies on these proteins.

Here we describe a novel system in which it is possible to produce N-terminally acetylated recombinant proteins from bacteria. By co-expressing the fission yeast NatB acetylation complex together with the target NatB substrate protein we have been able to acetylate and purify proteins from within *E. coli*. We went on to show that the same expression system works for each of the three N-terminal recognition sequences and successfully expressed acetylated human (Tropomyosin and Spartin) and yeast proteins (*S. pombe* Cdc8 and *S. cerevisiae* Tfs1). This simple and reliable methodology has the potential to allow significant savings in time and money over current techniques for generating amino-terminally acetylated recombinant polypeptides for both research and industrial applications.

## Results

**Figure 1:**
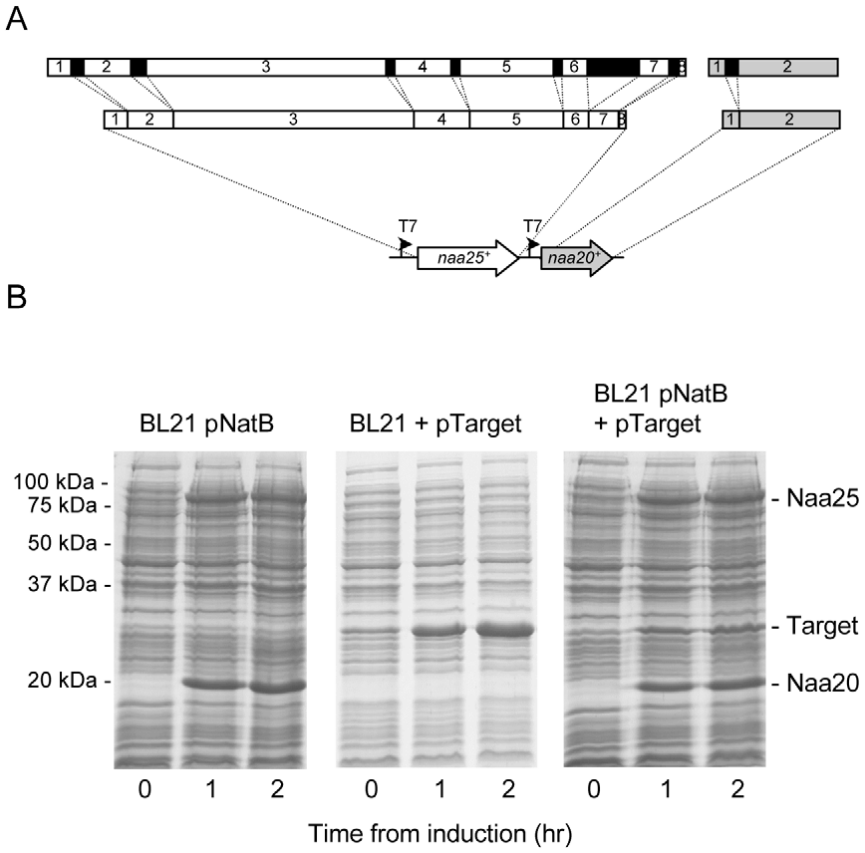
Generation of an *E.coli* NatB complex co-expression strain. (A) Introns (black regions) were removed from genes encoding the NatB subunits Naa20 (white) and Naa25 (grey), and the subsequent cDNAs were each cloned into the same bacterial expression vector (pNatB). (B) Whole cell lysates from BL21-DE3 cells containing either pNatB alone (left), pTarget (encoding the target protein alone - middle), or both pNatB & pTarget (right) were separated by SDS-PAGE following IPTG induction and visualised using coomassie stain. These data confirmed the successful co-expression of the NatB complex and target proteins in *E. coli*.

Using a system based on the fission yeast NatB acetylation complex (Figure 1A), we have developed a method in which these are co-expressed together with target substrates in *E. coli* (Figure 1B). NatB conjugates an acetyl group on the amino terminal methionine of peptides with Met.Asp, Met.Glu or Met.Asn as N-terminal sequences and therefore acetylate a significant proportion of eukaryotic proteins [8].

Mammalian striated muscle α-Tropomyosin (Tm) is an evolutionarily conserved actin binding protein that is a substrate for the NatB complex [9], [10]. This protein is readily expressed and purified from *E. coli* [11], and since the amino-terminal acetylation is essential for actin binding [11] it provides a simple test system for successful acetylation.

**Figure 2:**
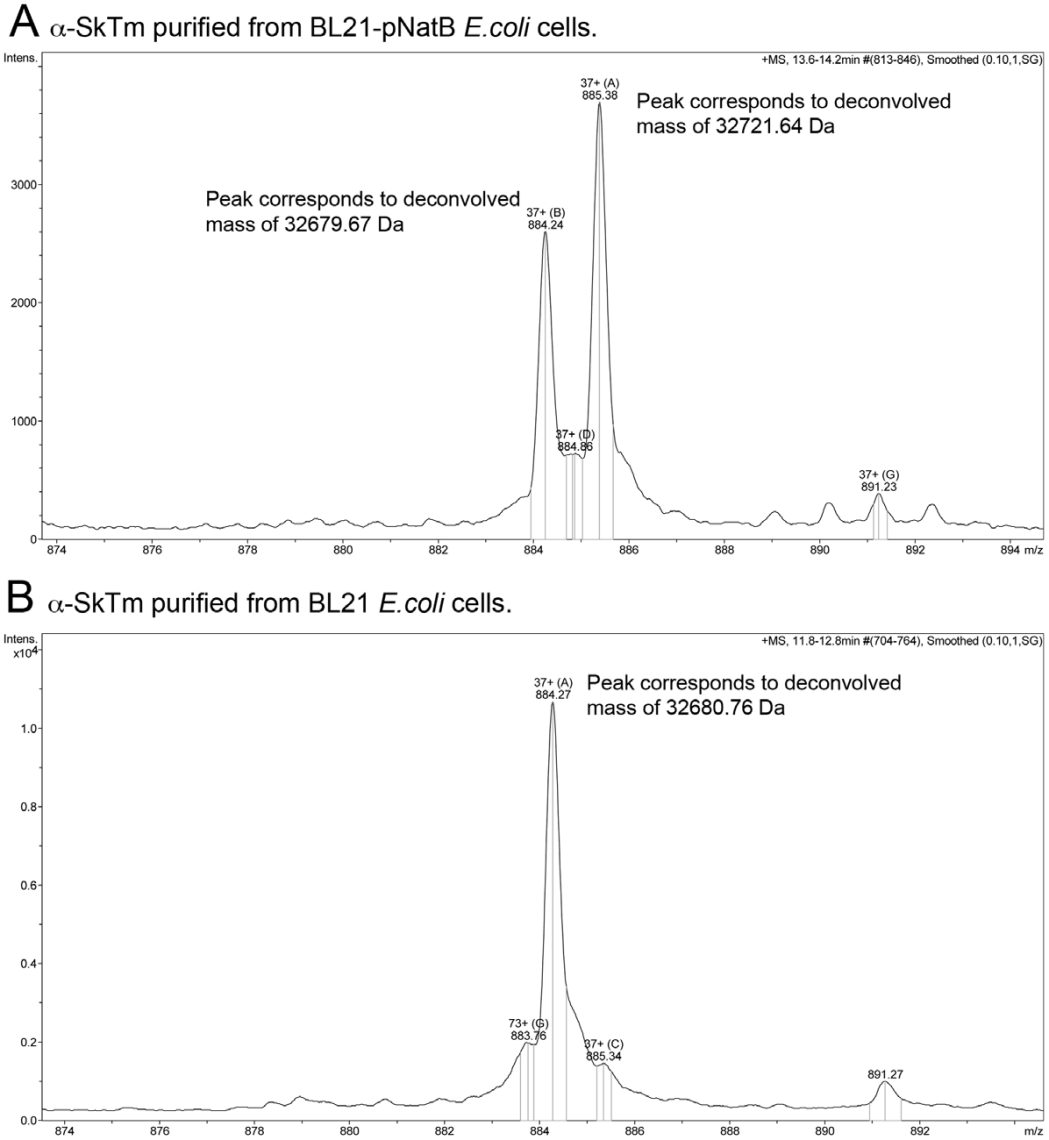
Electron-spray mass-spectroscopy spectra for α-SkTm. α-SkTm tropomyosin was purified from either *E. coli* BL21-pNatB cells (A) or *E. coli* BL21 cells (B). The undeconvolved mass charge envelope spectra show the purity of the proteins. Deconvolution of these data indicates that while ∼60% of the α-SkTm purified from BL21-pNatB cells is acetylated (additional 42 daltons mass), all of the Tm purified from standard BL21 cells remains unacetylated.

**Figure 3:**
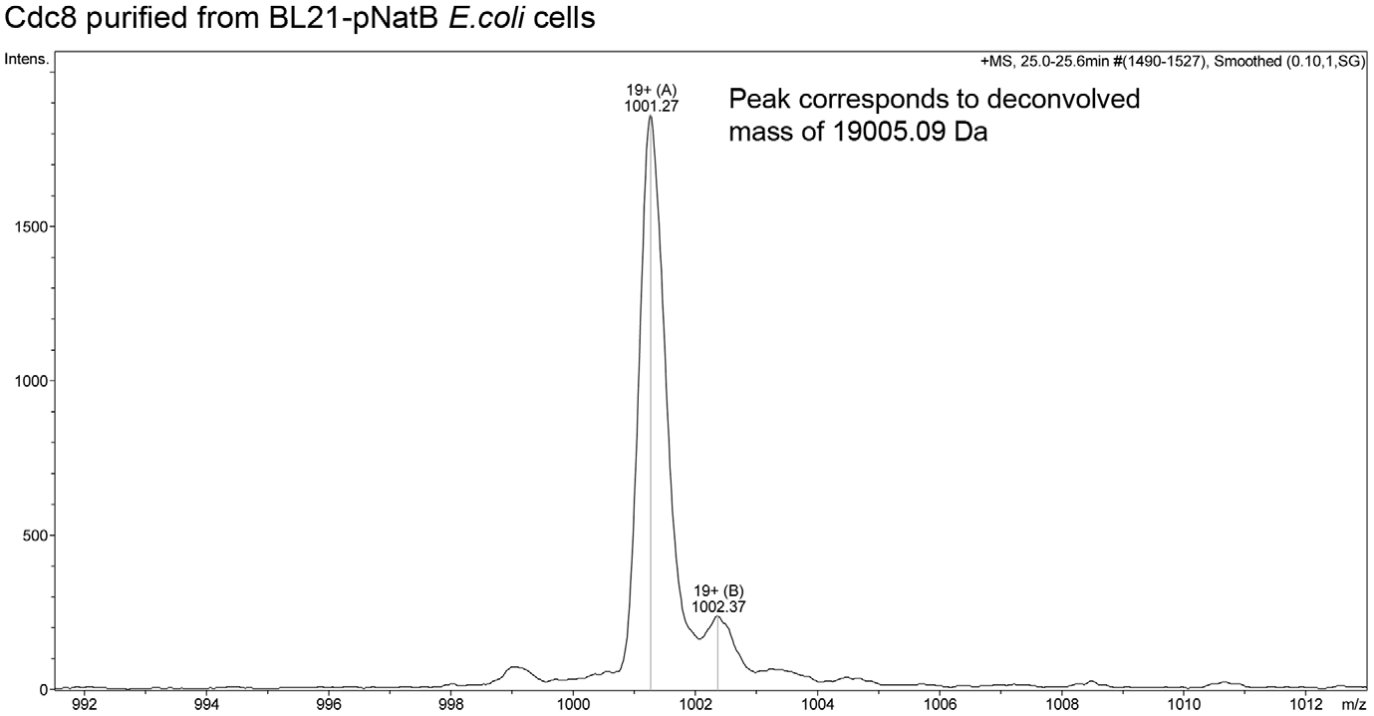
Electron-spray mass-spectroscopy spectra for *S. pombe* Cdc8. Cdc8 was purified from *E. coli* BL21-pNatB cells. The un-deconvolved mass charge envelope spectra show the purity of the protein. Deconvolution of this data indicates that all of the Cdc8 purified from BL21-pNatB cells is acetylated.

We co-transformed *E.coli* cells with the plasmid containing cDNA encoding for both catalytic (Naa20) and regulatory (Naa25) subunits of the fission yeast NatB complex, together with a plasmid encoding the murine α-skeletal Tm gene. These genes were co-induced (Figure 1B) and the Tm was isolated. Tm was purified and mass spectroscopy analysis shown that >60% of the Tm had been successfully acetylated (Figure 2A). This is in contrast to Skeletal Tm purified from standard BL21 cells which remained unacetylated (Figure 2B). The acetylated protein was shown, using an actin sedimentation assay, to bind actin with an affinity equivalent to the native protein [12], whereas the non acetylated protein failed to bind at Tm concentrations of up to 20 µM (Table 1). The muscle Tm has the N-terminal sequence Met-Glu. We went on to acetylate further proteins to confirm this prokaryote acetylation system worked for proteins with each of the NatB recognition sequences. Functional fission yeast Tm (Cdc8, N-terminal: Met.Asp) [6] was successfully produced (Figure 3) and bound to actin filament six times tighter than unacetylated yeast Tm [6]. Both human Spartin [6] (N-terminal: Met.Glu) and the budding yeast Tfs1 [13] (N-terminal: Met.Asn) proteins were also successfully acetylated and purified using affinity chromatography (data not shown) from *E. coli* (Table 1).

**Table 1:** Yield, acetylation efficiency and actin affinity of target proteins heterologously expressed in *E. coli*.

Protein	Host	N-term	% Acetylated in BL21 pNatB*	Protein Yield^3^		K_D_ for actin	
				- NatB	+ NatB	- NatB	+ NatB
Cdc8 Tm	*S. pombe*	M-D-	100%^1,2^	8.8	19.2	2.76 µM	0.45 µM
Skeletal Tm	*M. musculus*	M-E-	60%^1,2^	9.36	20.22	>20 µM	0.6 µM
Tfs1	*S. cerevisiae*	M-N-	30%^1,2^	1.6	5.4	-	-
Spartin	*H. sapiens*	M-E-	25%^2^	0.315	0.332	-	-

*determined by mass spectroscopy

1 1 or 2-dimensional gel electrophoresis2

2 ;

3 mg purified protein/litre of culture.

Three of the four target proteins expressed (SkTm, Cdc8, Tfs1) showed a 2–3 fold increase in yield when co-expressed with the NatB complex compared to standard expression systems. Whether this reflects an increase in the stability of the acetylated protein is unclear, and is currently under investigation. However the level of acetylation was variable from 25–100% for the four proteins. This may be improved by temporal control of expression to ensure NatB is both expressed and functional before the target protein is expressed. We have not yet examined whether the endogenous *E. coli* proteins were acetylated in the BL21-NatB cells, but if this was the case there was no associated detrimental effect upon cell morphology or growth.

## Discussion

Nat complex dependent amino-terminal acetylation has been assumed to occur on the eukaryote ribosome. If this were to be the case then our result would demonstrate that NatB is able to function at either the pro- or eukaryotic ribosome. However, as the composition of the bacterial and eukaryote ribosomes differ significantly it is uncertain how and where NatB functions within the bacterial cell, and is under further investigation.

The efficient and simple method we described in this paper has the potential to allow significant savings in time and money over current methods for chemically acetylating amino termini of many proteins and peptides, and also in improving yield during recombinant protein production. The ability to produce acetylated protein will now allow more detailed studies of the role of protein acetylation in protein stability, regulation of structure and function, as well as allowing the generate low cost proteins and peptides for the biotechnology & pharmaceutical industries.

## Materials and Methods

### Molecular Biology

*naa20^+^* (SPCC16C4.12) and *naa25^+^* (SPBC1215.02c) genes were amplified from genomic *S. pombe* DNA as *Nde1-BamH1* and *Sal1* fragments respectively and cloned into pGEM-T-Easy (Promega). Introns were removed sequentially by ligating blunt ended products of PCR reactions where appropriate primers had been used to amplify the entire plasmid lacking individual introns. Subsequent cDNAs were sequenced and cloned into pACYCduet (Novagen), each under the control of separate T7 promotors, to generate pACYCduet-*naa20^+^*-*naa25^+^* (pNatB). BL21 DE3 cells were co-transformed with pACYCduet-*naa20^+^*-*naa25^+^* and a pJC20 plasmid containing the cDNA encoding for the appropriate target protein also under the control of the T7 promoter (pTarget).

### Cell Culture

*E. coli* cells were cultured in NZY medium (1.0% Casein hydolysate (NZ amine), 0.5% NaCl, 0.5% yeast extract, 20 mM D-Glucose, 12.5 mM MgCl_2_ and 12.5 mM MgSO_4_) supplemented with appropriate antibiotics, and were grown in baffled Erlenmeyer flasks at 37°C with vigorous shaking. T7 dependent expression was induced by addition of IPTG (100 µg/ml final concentration) once cell cultures had reached an OD_600_ of 0.4–0.5. Cells were harvested 4 hr after induction with IPTG. Protein expression was assessed by separating whole cell lysates using SDS-PAGE and visualizing proteins with Coomassie blue stain.

### Biochemical techniques

Tropomyosin proteins were expressed and purified as described previously [6], while poly-histidine tagged proteins were purified on nickel columns (Qiagen) in denaturing conditions (8 M urea, 0.1 M NaH_2_PO_4_ 0.01 M Tris-Cl). Protein concentrations were determined using 280 nm extinction coefficients of 2,980 cm^−1^, 27,600 cm^−1^, 27,550 cm^−1^ and 64,070 cm^−1^ for Cdc8, α-SkTm, Tfs1 and Spartin respectively. Protein mass was determined using a Finnegan Mat LCQ ion-trap mass spectroscope. Cosedimentation assays were performed at 25°C as described previously [14]. We made use of the fact that acetylated SkTm migrates separately to the unacetylated form on SDS-PAGE to determine the K_D_ for of the acetylated population of Tm.

## References

[1] Arnesen T, Van Damme P, Polevoda B, Helsens K, Evjenth R (2009). Proteomics analyses reveal the evolutionary conservation and divergence of N-terminal acetyltransferases from yeast and humans.. Proc Natl Acad Sci U S A.

[2] Groenen PJ, Merck KB, de Jong WW, Bloemendal H (1994). Structure and modifications of the junior chaperone alpha-crystallin. From lens transparency to molecular pathology.. Eur J Biochem.

[3] Wada M, Shirahata A (2010). Identification of the primary structure and post-translational modification of rat S-adenosylmethionine decarboxylase.. Biol Pharm Bull.

[4] Kikuchi J, Iwafune Y, Akiyama T, Okayama A, Nakamura H (2010). Co- and post-translational modifications of the 26S proteasome in yeast.. Proteomics.

[5] Mannherz HG, Mazur AJ, Jockusch B (2010). Repolymerization of actin from actin:thymosin beta4 complex induced by diaphanous related formins and gelsolin.. Ann N Y Acad Sci.

[6] Skoumpla K, Coulton AT, Lehman W, Geeves MA, Mulvihill DP (2007). Acetylation regulates tropomyosin function in the fission yeast *Schizosaccharomyces pombe*.. J Cell Sci.

[7] Gautschi M, Just S, Mun A, Ross S, Rucknagel P (2003). The yeast N(alpha)-acetyltransferase NatA is quantitatively anchored to the ribosome and interacts with nascent polypeptides.. Mol Cell Biol.

[8] Polevoda B, Arnesen T, Sherman F (2009). A synopsis of eukaryotic Nalpha-terminal acetyltransferases: nomenclature, subunits and substrates.. BMC Proc.

[9] Polevoda B, Cardillo TS, Doyle TC, Bedi GS, Sherman F (2003). Nat3p and Mdm20p are required for function of yeast NatB Nalpha-terminal acetyltransferase and of actin and tropomyosin.. J Biol Chem.

[10] Singer JM, Shaw JM (2003). Mdm20 protein functions with Nat3 protein to acetylate Tpm1 protein and regulate tropomyosin-actin interactions in budding yeast.. Proc Natl Acad Sci U S A.

[11] Urbancikova M, Hitchcock-DeGregori SE (1994). Requirement of amino-terminal modification for striated muscle alpha-tropomyosin function.. J Biol Chem.

[12] Boussouf SE, Maytum R, Jaquet K, Geeves MA (2007). Role of tropomyosin isoforms in the calcium sensitivity of striated muscle thin filaments.. J Muscle Res Cell Motil.

[13] Caesar R, Blomberg A (2004). The stress-induced Tfs1p requires NatB-mediated acetylation to inhibit carboxypeptidase Y and to regulate the protein kinase A pathway.. J Biol Chem.

[14] Maytum R, Geeves MA, Konrad M (2000). Actomyosin regulatory properties of yeast tropomyosin are dependent upon N-terminal modification.. Biochemistry.

